# Bone Remodeling and Marginal Bone Loss of Simplified Versus Conventional Drilling: A Randomized Clinical Trial

**DOI:** 10.3390/bioengineering12020178

**Published:** 2025-02-13

**Authors:** Alberto Ruiz García, Artiom Lijnev, Fatemeh Soleymani, Jeevithan Elango, José Eduardo Maté Sánchez de Val, Carlos Pérez-Albacete Martínez

**Affiliations:** 1 Health Sciences PhD Program, Universidad Católica de Murcia UCAM, Campus de los jerónimos 135, 30107 Murcia, Spain; albertoruizgarcia@hotmail.com; 2 Department of Biomaterials Engineering, Faculty of Health Sciences, Universidad Católica de Murcia UCAM, Campus de los jerónimos 135, 30107 Murcia, Spain; fsoleimani@ucam.edu (F.S.); jelango@ucam.edu (J.E.); jemate@ucam.edu (J.E.M.S.d.V.); cperezalbacete@ucam.edu (C.P.-A.M.)

**Keywords:** drilling protocol, marginal bone loss, simplified drilling protocol, implant survival, implant torque

## Abstract

This study evaluates the influence of conventional versus simplified drilling protocols on bone remodeling after the osteointegration period, marginal bone loss (MBL), and primary implant stability. A randomized, double-blind clinical trial was conducted involving 44 implants in 37 patients over a two-year period. The primary outcome was peri-implant tissue stability, measured as MBL at baseline, 12 months, and 24 months. Secondary outcomes included implant stability, measured via insertion torque, and survival rates. The results indicated no significant differences in initial bone remodeling and MBL between groups after 24 months. Both protocols demonstrated high survival rates, with one implant failure recorded in the simplified protocol group. Although simplified drilling protocols may reduce surgical complexity, concerns about heat generation and reduced adaptability in osteotomy were described in the literature. This study concludes that drilling protocol choice does not significantly impact bone levels during osteointegration, crestal bone maintenance, or implant survival over 24 months, but further research is needed to explore long-term effects and prosthetic factors.

## 1. Introduction

In contemporary dental implantology, significant strides have been made in achieving reliable osseointegration and long-term survival. Despite these advancements, marginal bone loss (MBL) around dental implants remains a critical issue, particularly concerning the long-term stability and health of peri-implant tissues [1,2]. As implant procedures become more common worldwide, the focus has shifted to identifying surgical techniques that can mitigate the risk of MBL. Among these, the drilling protocol used during implant placement has emerged as a key factor in influencing MBL outcomes [3].

Traditionally, conventional drilling protocols, involving the sequential use of drills with increasing diameters, have been widely accepted. While effective, concerns have been raised about the potential bone trauma caused by the mechanical and thermal stresses of this approach [4,5]. These stresses have been linked to early MBL, particularly during the critical healing period following implant placement [6]. As a result, alternative approaches, such as undersized drilling, piezoelectric implant site preparation (PISP), and osseodensification drills (ODs) have been described, with each designed to decrease the risks associated with traditional methods [7,8,9,10].

In addition to these approaches, simplified drilling protocols have gained attention for their potential to reduce surgical complexity while maintaining peri-implant bone health [11]. Preclinical studies have shown that these protocols, by reducing the number of drill steps, can decrease mechanical and thermal trauma during implant placement, which may lead to lower rates of bone resorption, making them a viable alternative to conventional methods [12,13].

Despite the growing interest in these alternative drilling protocols, there is a lack of robust clinical evidence evaluating their long-term effects on MBL. 

The aim of the present study was to evaluate the effect of implant site preparation techniques (conventional drilling versus simplified) on marginal bone level during the osteointegration period and after prosthetic delivery; additionally, insertion torque changes were evaluated, while also assessing whether a simplified drilling protocol can achieve clinical outcomes comparable to those of the conventional full-sequence drilling approach. With increasing experience in the field, there is a growing inclination to streamline procedures by focusing on their core principles. This approach not only seeks to enhance procedural efficiency but also addresses concerns related to time savings and material utilization.

The null hypothesis posits that there is no significant difference in marginal bone levels during the osteointegration period and marginal bone loss (MBL) between the simplified drilling protocol and the conventional full-sequence drilling protocol.

## 2. Materials and Methods

### 2.1. Trial Design, Participants, and Settings

This study was designed as a balanced, randomized, double-blinded clinical trial, which was conducted with two parallel experimental arms: test and control. The participants were recruited solely from Spain. The trial was conducted from December 2020 to July 2023. The study was approved by the Regional Committee for Research Ethics (Ref. CE092002) and was registered in ClincalTrials.gov under number NCT06780956.

Patients who met the inclusion criteria were recruited by the Unit of Oral Surgery and Implantology of the Catholic University of Murcia from December 2020 to July 2023. The patients were fully informed of the characteristics of the study and were invited to participate. A complete medical history was taken for each patient, and they also underwent a thorough oral examination and a cone beam tomography (CBCT)-based radiology study (Dental Sirona). The tests were carried out in accordance with the criteria recommended in the CONSORT Guidelines.

The selection criteria for this study were as follows: (1) patients without any systemic pathologies that could be considered as grounds for absolute contraindication; (2) adult patients who agreed to form part of the study and who signed the informed consent form; (3) patients who consumed less than ten cigarettes/day; (4) patients who were not completely edentulous; (5) patients partially edentulous in the posterior maxillary or mandibular area that did not require the use of regenerative techniques; and (6) patients with an area of healed mature bone at least 3 months post-extraction. Subjects were not included in the RCT if any of the following exclusion criteria were met: (1) patients lacking teeth in esthetic zones 13–23 and 33–43 (second and fifth sextants); (2) patients who smoked more than ten cigarettes a day; (3) patients with a bleeding index of more than 30%; (4) patients with dental caries or periodontal diseases; and (5) pregnant or lactating women. In other cases of patients who required multiple dental replacements, implants could be placed next to each other as long as they met the randomization criteria.

This was a preliminary 2-year report as part of an ongoing study. It was planned to follow-up patients to the fifth year post-loading in order to evaluate the effects of the mentioned procedures over time.

### 2.2. Interventions

Patients were divided into two parallel experimental arms. The arms were the conventional drilling protocol and the simplified drilling protocol. Prior to surgical interventions, detailed case planification was performed, and implant size was selected according to safety margins (1.5 mm from buccal bone, 1 mm from lingual bone, and 1 mm from inferior alveolar nerve). The drilling protocol was dependent on the bone quality, which was classified as type I (dense) bone, type II and III (medium) bones, and type IV (soft) bones, and the manufacturer’s instructions were followed for this purpose.

#### 2.2.1. Study Products

**Figure 1:**
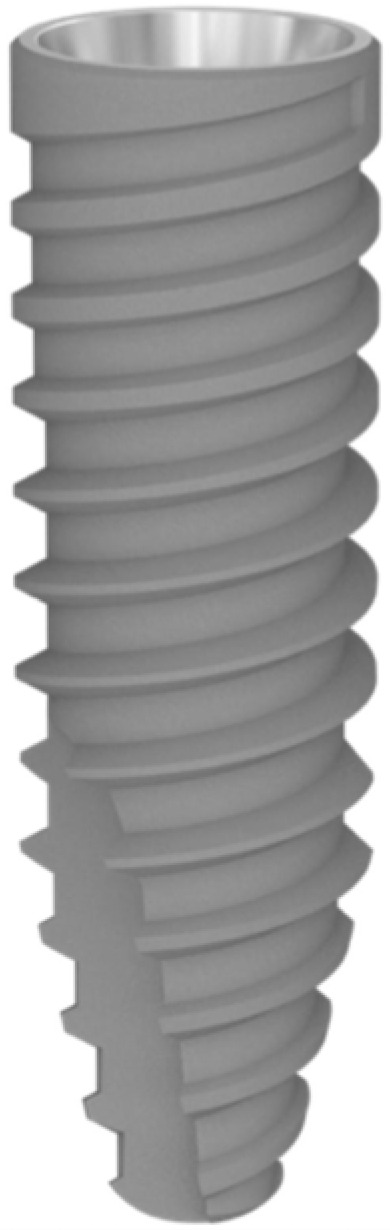
The implant macrodesign features utilized in the study.

Forty-six TOP DM^®^ Hexagonal Internal Connection Implants (Bioner, Sistemas Implantológicos, Barcelona, Spain) (Figure 1), each with a diameter ranging from 3.5 to 5 mm, were placed in healed mature bone (more than 3 months post-extraction) as well as 46 DM-PC screw-retained healing abutments (Bioner, Sistemas Implantológicos, Barcelona, Spain).

**Figure 2:**
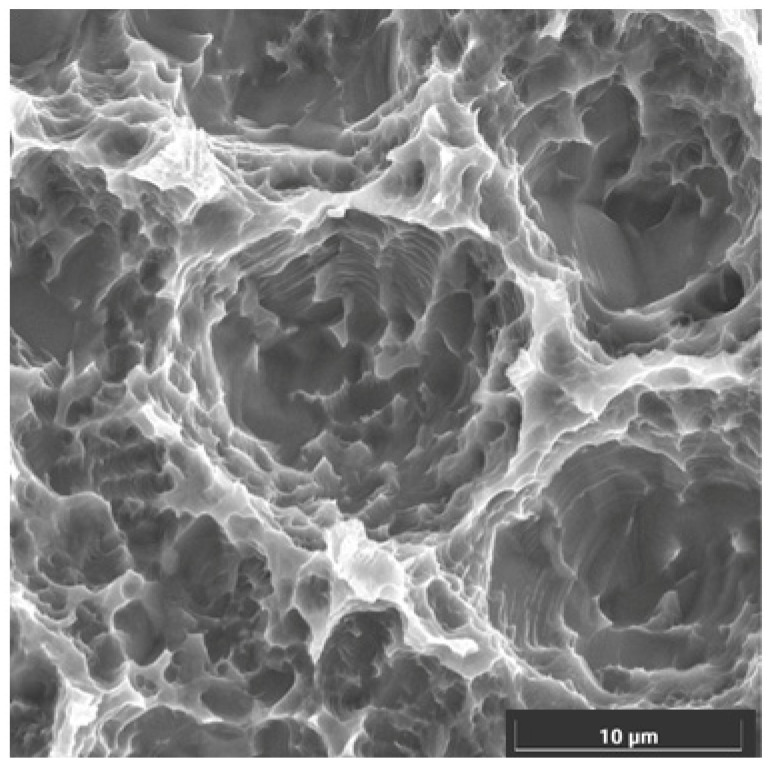
SEM image of surface characteristics of implants.

This implant model was manufactured from Ti-V, and it boasted a macroscopic design, which favors primary stability in any situation. The implant model had an internal conical connection and a single prosthetic platform. Microscopically, it had a biotechnological surface treated with a double acid attack and an average roughness (Ra) of 1.3 μm (Figure 2).

#### 2.2.2. Surgical Procedures

In the control group (conventional protocol), the implants were placed following the routine surgical technique for bone-level implants with a mucoperiosteal flap. Drilling the implant bed was performed according to the manufacturer’s instructions. Briefly, after the initial lance drill combined with a pilot drill to mark the direction of insertion, bone quality was evaluated. After evaluation, and depending on the type of bone, the protocol consisted of increasing the drill diameters according to the hardness of the bone. The implants were placed mechanically up to a maximum of 70  Ncm, and the implantation process was finished manually using a surgical torque wrench. It was determined that the implant should always be placed below the residual alveolar crest.

**Figure 3:**
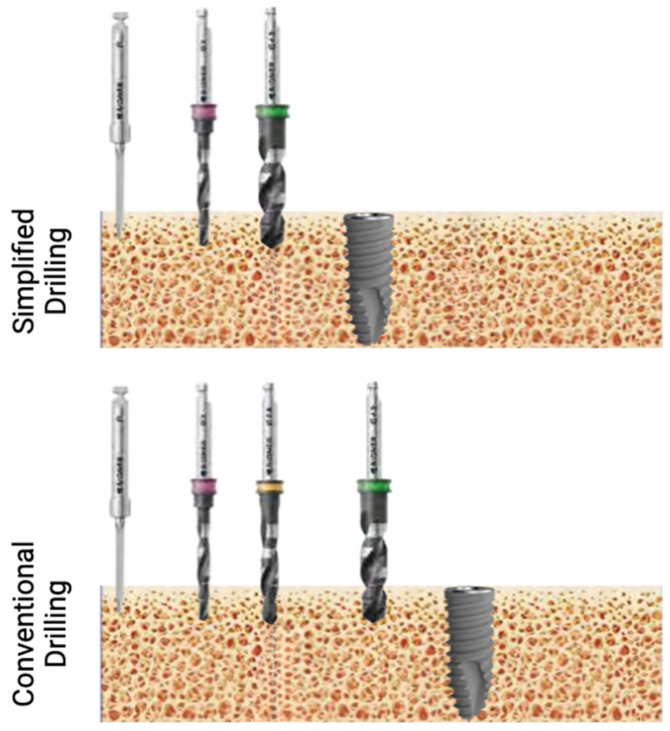
Schematic image comparing simplified and conventional drilling protocols in type III–IV bone for implants with a diameter of 4 mm.

In the test group (simplified protocol), after local anesthesia and mucoperiosteal flap elevation, the simplified drilling protocol was applied, consisting of a lance drill, pilot drill, and final drill. The final drill procedure was selected according to the bone quality. Then, the prepared final implants were placed and sutured (Figure 3).

After the surgical interventions, the patients were instructed to proceed with prophylactic antibiotic therapy, and 600 mg ibuprofen tablets were prescribed as an anti-inflammatory to be taken three times a day, as long as required. The patients were instructed to use 0.2% chlorhexidine mouthwash for 1 min twice a day for 2 weeks and to avoid brushing and trauma to the surgical sites. No removable prosthesis was allowed. Sutures were removed after 14 days, and oral hygiene instructions were given and reinforced at control and measurements visits. Three-dimensional radiological examinations were performed at the following time points: initially during treatment planning, after implant placement, at 3 months during prosthetic delivery, and at 12- and 24-month follow-up periods.

#### 2.2.3. Healing Abutment Insertion

All the abutments were placed after a healing period of 3 months. The implants were recovered by crestal incision and sutured after placement. The abutments were screwed at a minimum of 20  Ncm.

#### 2.2.4. Definitive Prosthesis

The impressions for the definitive prosthesis and its placement were taken 2 weeks after the healing abutment insertion. The metal–porcelain prosthesis was screwed to the definitive abutment using a burnout cap, and the torque used for the definitive prosthesis was 20  Ncm.

### 2.3. Measurements and Primary and Secondary Outcomes

The main outcome was marginal bone remodeling following different drilling protocols during an osteointegration period of 3 months and peri-implant tissue stability, measured as MBL using three-dimensional digital radiology (Orthophos SL 3D, Dental Sirona) at restoration delivery up to 12 and 24 months after baseline measurement.

The secondary outcomes were as follows: (1) Primary stability of the implants at insertion evaluated by means of Ncm on a manual wrench. (2) Implant survival rate after 24 months of loading, defined as implants not being lost regardless of condition.

Radiological acquisitions were obtained using the same three-dimensional radiology device (Orthophos SL 3D, Dental Sirona, Charlotte, NC, USA) and were visualized using digital software (Sidexis 4, Dental Sirona). To calculate marginal bone remodeling, the initial implant position was established as the baseline, and after an osteointegration period of 3 months, the restorative phase was counted as the final period of the remodeling period. In order to calculate MBL, a calibration calculation based on the known diameter of the implant and/or the implant height was first performed for each implant. The position of the implant neck in relation to the most coronal portion of the peri-implant bone crest was taken as a reference point for the implants. MBL was calculated as the difference between the bone position values measured during two periods in the mesial and distal areas of each implant.

The measurements were taken by one examinator (A.R.G). The calibration exercise was completed prior to the study in the Oral Surgery Unit using 10 patients who underwent treatment in this unit but who were not part of the study. The reliability of the measurements performed by the one examiner at different time points was evaluated using the intra-class correlation coefficient for MBL. Values above 0.80 were considered as having a high degree of reliability in terms of the measurements.

### 2.4. Sample Size

A sample size calculation was established based on previous studies [14]. By assuming an effect size (ES) of 0.5 mm on MBL, an alpha error of 0.05, a statistical power of 80%, and an estimated loss ratio of 20%, 23 implants in each group, 46 implants in total, were determined.

### 2.5. Randomization (Random Number Generation, Allocation Concealment, and Implementation)

**Figure 4:**
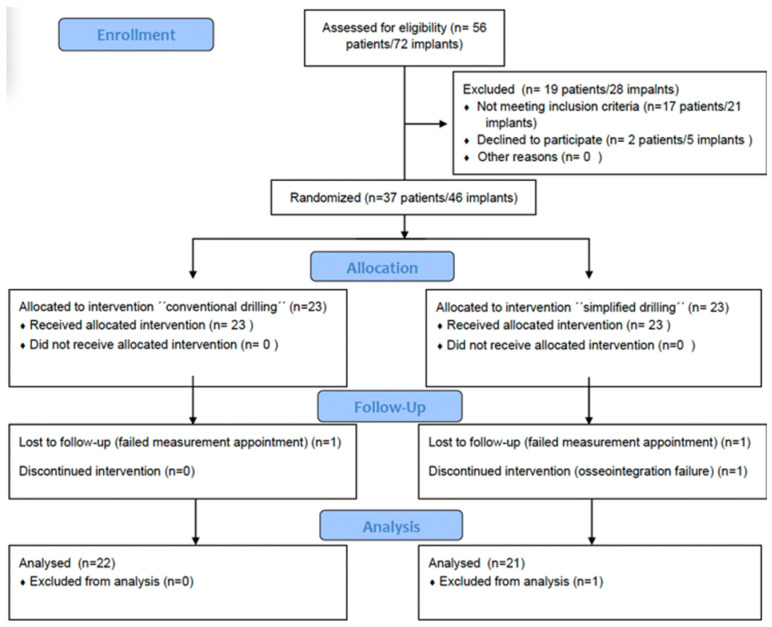
Schematic image of random number generation, allocation concealment, and implementation.

As patients were recruited prior to the surgical procedure, implants were randomized by means of block randomization, ensuring an equal number distribution among the drilling groups for implant placement using an SPSS 24.0 macro (Figure 4).

### 2.6. Blinding

This was a randomized, double-blind clinical trial, in which the patients and the surgeon performing the implant placement were unaware of the group that each subject had been assigned to. In order to ensure surgeon blinding, once the mucoperiosteal flap was elevated, an envelope with patient allocation containing the drilling protocol was opened and followed.

### 2.7. Statistical Analysis

The analysis used the implant as the main unit of study. The continuous variables were expressed as mean  ±  SD, and the categorical variables were expressed as frequencies and percentages. The samples were checked for normality using the Shapiro–Wilk test. The independent sample *t*-test was used to compare the means between the two study groups. The Pearson chi-square (or likelihood ratio) test was used to compare the categorical variables. In the survival analysis, the Kaplan–Meier method was used for calculating the survival of the implants. The statistical analysis was performed using the IBM SPSS 24.0 software (IBM Inc., New York, NY, USA), and the significance level was established at *p* < 0.05. The effect of the treatment on the primary outcome was assessed by constructing multiple linear regression models, considering MBL as the dependent outcome and group, gender, implant diameter, implant length, arch, and substituted tooth as independent variables.

## 3. Results

### 3.1. Sample Description

Of the 46 initial implants, 2 were excluded from the study due to failed revision and measurement appointments, and one discontinued the intervention due to osseointegration failure. The final sample consisted of 44 implants that were placed in 37 patients, 25 men (57%) and 19 women (43%). A total of 22 (50%) had their implants placed using the conventional drilling protocol, and the other 22 (50%) had their implants placed using the simplified protocol. The average age of the patients was 53.2  ±  13.4, with a range from 20.7 to 76.8 years. A summary of all the variables of the study is presented in Table 1 and Table 2.

### 3.2. Bone Levels at Implant Placment and Restoration Delivery

Following the subcrestal implant placement, the position of the implants was established as the baseline for the evaluation of bone remodeling during the osteointegration period. Both groups did not present statistically significant differences at the bone levels at this point, with a difference in the average of 0.1 mm between the groups (*p* = 0.688).

At restoration delivery, the position of the implants was recorded as the starting point for further measurements to evaluate MBL. Both groups had their implant platform positioned subcrestally. In the mesial area, the implants were placed less subcrestally in the conventional protocol, with no statistical difference between the groups (*p* = 0.372). In the distal area, the bone level at restoration delivery was more subcrestal in the conventional drilling protocol (*p* = 0.027).

### 3.3. Bone Remodeling Following Osteointegration Period

The major changes in the bone levels were observed during the post-osteointegration period. The effects of the drilling protocol on the physiological process of initial bone remodeling following the implant insertion were not significant. After a 3-month period, both protocols experienced similar average changes in bone levels, i.e., 1.24 mm and 1.25 mm (*p* = 0.98) for the conventional and simplified protocols, respectively.

### 3.4. Marginal Bone Loss (MBL) at 12 and 24 Months After Restorative Period

The early and 24-month marginal bone loss (MBL) was evaluated to assess the differences between the two drilling protocols. The early MBL was statistically significant between the two groups, with the distal and average MBL values showing significant differences (*p* = 0.022 and *p* = 0.026, respectively) (Table 3). Over the 24-month follow-up period, the implants demonstrated great stability, with an average MBL of 0.13 (0.41) for the conventional drilling protocol and 0.28 (0.71) for the simplified drilling protocol (*p* = 0.413). No statistical differences were found in the mesial or distal aspects of the implants (*p* = 0.662 and *p* = 0.363, respectively).

The treatment effect, in terms of MBL, was assessed by multiple regression models, incorporating several factors as potential sources of variability (Supplementary Material Table S1). The final model could explain 25.8% of the variability (*p*-value = 0.130) in the average MBL from the baseline to 2 years follow-up, with gender (*p* = 0.032) demonstrating a significant effect on the primary outcome.

### 3.5. Primary Stability

The average insertion torque (Ncm) of the implants was 46.82  ±  14.89  Ncm, with a range of 10–70  Ncm, and no differences were observed when taking the drilling protocol into account (*p* = 0.705).

### 3.6. Survival Analysis

**Figure 5:**
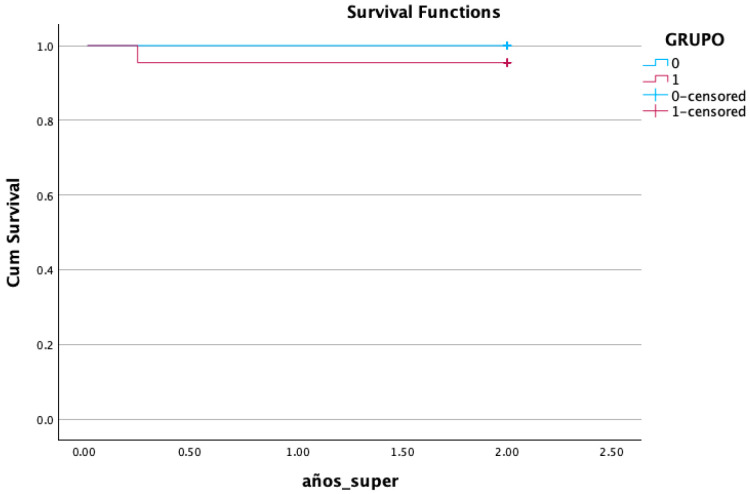
Kaplan–Meier survival analysis.

Implant failure occurred in one case (2.3%) at the uncovering period after placement corresponding to the simplified drilling protocol. The early implant failure could be related to the insertion torque being less than 15 Ncm at implant placement due to oversized bed preparation (Table 4) (Figure 5).

## 4. Discussion

Minimizing MBL and promoting implant stability should be one of the objectives of all professionals who perform dental implant treatments, given that these facilitate peri-implant health maintenance. For a large part of dental implant history, the mechanisms behind MBL have been barely understood. However, recent research has thrown light upon this topic, and clinicians are now aware of several local conditions among patients, implant system design characteristics, and technical (surgical/prosthetic) aspects that have an impact on MBL loss [15].

This randomized clinical trial aimed to compare physiological bone remodeling following implant insertion, MBL, and primary stability of implants inserted into sites prepared with conventional and simplified drilling protocols. Being one of the few clinical studies to directly compare these two types of drilling protocols, it was possible to examine and discuss only the available evidence resulting from studies in which these two types were used. The results from the present study suggest that MBL at a period of 12 and 24 months after loading was not affected by the drilling protocols.

The results are in accordance with previous studies, where simplified drilling protocols using only one drill did not demonstrate superiority, although most studies did not provide a long term follow-up [11,16]. Another factor to be considered is that previous studies focused on MBL using two-dimensional radiographic images; the advantage of evaluating MBL with three-dimensional radiography relies on height the sensitivity of the measurements.

In terms of primary stability, which is related to the overall success of osseointegration, our study could not prove the superiority of this treatment modality, with it showing no statistical difference; these results may be compared to other studies where similar findings were found using different drilling protocol modalities [16,17,18].

After 12 months and 24 months of prosthetic loading, 43 out of the 44 implants were satisfactorily functioning. One implant failed in the simplified protocol group before loading (11/22 95.5% survival rate), while no failures were recorded in the conventional protocol group (0/22; 100% survival rate). Also, for this outcome, no significant differences were demonstrated between the two groups, in accordance with previous clinical studies and meta-analyses reporting similar survival rates for implants inserted with different implant site preparation techniques [6,18,19,20].

Another aspect to evaluate is the time implied to prepare the osteotomy bed. Some studies suggest that by performing a short drilling protocol, the clinician would be able to reduce the timing by 50% compared to a full-sequence drilling protocol; however, some limitations have been reported, for example, the possibility of correcting the implant position during a staged osteotomy is significantly reduced; thus, this simplified protocol is recommended to more experienced surgeons [11,16].

One of the potential drawbacks that may reduce its clinical use is the higher heat generation during the simplified preparation protocol. Some studies have confirmed that in order to overcome the heat increase, the addition of a pilot drill before the final drill must be considered, especially in dense bone types. Additionally, scopious saline irrigation during bed preparation is required [21,22,23]. However, Sihana Rugova and Marcus Abboud found that under a standardized and unbiased drilling procedure in a similar bone density, the one-drill protocol caused less heat generation.

The main limitation of the present study was the lack of analysis of prosthetic and soft tissue factors that could potentially impair the results, which are recommended to be taken into account in future studies [24].

## 5. Conclusions

The main findings of the present study indicate no significant differences in bone remodeling after the osteointegration period and crestal bone maintenance (MBL) over 12- and 24-month periods, regardless of the drilling protocol applied. Furthermore, the drilling protocol does not appear to influence primary stability at the time of implant placement or survival rates. Future investigations should focus on longer follow-up periods and consider the impact of prosthetic factors to provide a more comprehensive understanding.

## Figures and Tables

**Table 1:** Baseline variables by the type of drilling protocol.

Variable	Conventional (%)	Simplified (%)	*p* Value
Gender			0.361 Pearson’s chi-square
Male	11 (50%)	14 (63.6%)	
Female	11 (50%)	8 (36.4%)	
Substituted tooth			1.00 Pearson’s chi-square
Premolar	7 (31.8%)	9 (31.8%)	
Molar	15 (68.2%)	13 (68.2%)	
Arch			0.540 Pearson’s chi-square
Maxilla	8 (36.3%)	10(45.4%)	
Mandible	14 (63.7%)	12 (54.6%)	
Implant insertion torque			0.487 (likelihood ratio)
<15 N	0 (0%)	1 (4.5%)	
15–40 N	10 (45.5%)	9 (40.9%)	
>40 N	12 (54.5)	12 (54.5%)	
Implant diameter			0.652 (likelihood ratio)
3.5	5 (22.7%)	6 (27.3%)	
4.0	14 (63.6%)	12 (54.5%)	
4.2	0 (0%)	1 (4.5%)	
5.0	3 (13.6%)	3 (13.6%)	
Implant length			0.106 (likelihood ratio)
7	0 (0%)	1 (4.5%)	
8.5	8 (36.4%)	2 (9.1%)	
10	8 (36.4%)	12 (54.5%)	
11.5	6 (27.3%)	7 (31.8%)	

**Table 2:** Descriptive data on insertion torque and bone levels.

Variable	Conventional Mean (*±SD*)	Simplified Mean (*±SD*)	*p*-Value
Age	56.35 (12.22)	50.11 (14.16)	0.125
Insertion torque (primary stability) (Ncm)	47.68 (16.50)	45.95 (13.41)	0.705
Bone level at implant placment			
Mesial	1.60 (0.19)	1.56 (0.15)	0.904
Distal	1.74 (0.23)	1.58 (0.15)	0.579
Avarage	1.67 (0.18)	1.57 (0.14)	0.688
Bone level at restoration delivery			
Mesial	0.30 (0.33)	0.42 (0.50)	0.372
Distal	0.58 (0.60)	0.20 (0.26)	0.027
Average	0.42 (0.38)	0.31 (0.34)	0.171

**Table 3:** Outcomes, presented as mean changes and standard deviation (mean (*SD*)), between baseline–12 moths and basline–24 months.

Outcome Variable	Control, N = 22 (Conventional Drilling)	Test, N = 22 (Simplified Drilling)	Mean Difference (95% CI)	*p*-Value
ΔBone levels post-osteintegration				
Mesial	1.29 (1.04)	1.14 (0.83)	0.28 (−0.73; 0.43)	0.608
Distal	1.20 (1.14)	1.36 (0.71)	0.29 (−0.43; 0.75)	0.595
Avarage	1.24 (1.01)	1.25 (0.68)	0.26 (−0.53; 0.54)	0.98
ΔMBL 12 months post-restorative				
Mesial	0.01 (0.29)	0.16 (0.48)	0.14 (−0.10; 0.39)	0.257
Distal	0.11 (0.38)	0.44 (0.51)	0.32 (0.04; 0.60)	0.022
Average	0.06 (0.25)	0.30 (0.40)	0.23 (0.03; 0.44)	0.026
ΔMBL 24 months post-restorative				
Mesial	0.12 (0.68)	0.22 (0.85)	0.10 (−0.30; 0.58)	0.662
Distal	0.15 (0.63)	0.34 (0.72)	0.19 (−0.22; 0.60)	0.363
Average	0.13 (0.41)	0.28 (0.71)	0.14 (−0.21; 0.51)	0.413

**Table 4:** Survival rates of implants according to drilling protocol.

Variable	Conventional (%)	Simplified (%)	*p* Value
survival	22 (100%)	21 (95.5%)	0.371

## Data Availability

The original data presented in the study are openly available in repositoria Universidad Católica San antonio de Murcia.

## Supplementary Materials

The following supporting information can be downloaded at: https://www.mdpi.com/article/10.3390/bioengineering12020178/s1, Table S1: Multiple regression models.

## References

[1] Lombardi T., Berton F., Salgarello S., Barbalonga E., Rapani A., Piovesana F., Gregorio C., Barbati G., Di Lenarda R., Stacchi C. (2019). Factors Influencing Early Marginal Bone Loss around Dental Implants Positioned Subcrestally: A Multicenter Prospective Clinical Study. J. Clin. Med..

[2] Galindo-Moreno P., Catena A., Perez-Sayans M., Fernandez-Barbero J.E., O’Valle F., Padial-Molina M. (2022). Early marginal bone loss around dental implants to define success in implant dentistry: A retrospective study. Clin. Implant. Dent. Relat. Res..

[3] Ragucci G.M., Giralt-Hernando M., Mendez-Manjon I., Canto-Naves O., Hernandez-Alfaro F. (2020). Factors Affecting Implant Failure and Marginal Bone Loss of Implants Placed by Post-Graduate Students: A 1-Year Prospective Cohort Study. Materials.

[4] Peker Tekdal G., Bostanci N., Belibasakis G.N., Gurkan A. (2016). The effect of piezoelectric surgery implant osteotomy on radiological and molecular parameters of peri-implant crestal bone loss: A randomized, controlled, split-mouth trial. Clin. Oral Implant. Res..

[5] Pellicer-Chover H., Penarrocha-Oltra D., Aloy-Prosper A., Sanchis-Gonzalez J.C., Penarrocha-Diago M.A., Penarrocha-Diago M. (2017). Comparison of peri-implant bone loss between conventional drilling with irrigation versus low-speed drilling without irrigation. Med. Oral Patol. Oral Cir. Bucal.

[6] Li X., Lin X., Guo J., Wang Y. (2020). The Stability and Survival Rate of Dental Implants After Preparation of the Site by Piezosurgery vs Conventional Drilling: A Systematic Review and Meta-Analysis. Int. J. Oral Maxillofac. Implant..

[7] Stocchero M., Toia M., Cecchinato D., Becktor J.P., Coelho P.G., Jimbo R. (2016). Biomechanical, Biologic, and Clinical Outcomes of Undersized Implant Surgical Preparation: A Systematic Review. Int. J. Oral Maxillofac. Implant..

[8] Canullo L., Penarrocha D., Penarrocha M., Rocio A.G., Penarrocha-Diago M. (2014). Piezoelectric vs. conventional drilling in implant site preparation: Pilot controlled randomized clinical trial with crossover design. Clin. Oral Implant. Res..

[9] Sierra-Rebolledo A., Tariba-Forero D., Rios-Calvo M.D., Gay-Escoda C. (2021). Effect of undersized drilling on the stability of immediate tapered implants in the anterior maxillary sector. A randomized clinical trial. Med. Oral Patol. Oral Cir. Bucal.

[10] Shanmugam M., Valiathan M., Balaji A., Jeyaraj Samuel A.F., Kannan R., Varthan V. (2024). Conventional Versus Osseodensification Drilling in the Narrow Alveolar Ridge: A Prospective Randomized Controlled Trial. Cureus.

[11] Bratu E., Mihali S., Shapira L., Bratu D.C., Wang H.L. (2015). Crestal bone remodeling around implants placed using a short drilling protocol. Int. J. Oral Maxillofac. Implant..

[12] Gil L.F., Sarendranath A., Neiva R., Marao H.F., Tovar N., Bonfante E.A., Janal M.N., Castellano A., Coelho P.G. (2017). Bone Healing Around Dental Implants: Simplified vs Conventional Drilling Protocols at Speed of 400 rpm. Int. J. Oral Maxillofac. Implant..

[13] Giro G., Tovar N., Marin C., Bonfante E.A., Jimbo R., Suzuki M., Janal M.N., Coelho P.G. (2013). The effect of simplifying dental implant drilling sequence on osseointegration: An experimental study in dogs. Int. J. Biomater..

[14] Al Amri M.D., Al-Johany S.S., Al Baker A.M., Al Rifaiy M.Q., Abduljabbar T.S., Al-Kheraif A.A. (2017). Soft tissue changes and crestal bone loss around platform-switched implants placed at crestal and subcrestal levels: 36-month results from a prospective split-mouth clinical trial. Clin. Oral Implant. Res..

[15] Oh T.J., Yoon J., Misch C.E., Wang H.L. (2002). The causes of early implant bone loss: Myth or science?. J. Periodontol..

[16] Guazzi P., Grandi T., Grandi G. (2015). Implant site preparation using a single bur versus multiple drilling steps: 4-month post-loading results of a multicenter randomised controlled trial. Eur. J. Oral Implantol..

[17] Stacchi C., Troiano G., Montaruli G., Mozzati M., Lamazza L., Antonelli A., Giudice A., Lombardi T. (2023). Changes in implant stability using different site preparation techniques: Osseodensification drills versus piezoelectric surgery. A multi-center prospective randomized controlled clinical trial. Clin. Implant. Dent. Relat. Res..

[18] Stanford C.M., Barwacz C., Raes S., De Bruyn H., Cecchinato D., Bittner N., Brandt J. (2016). Multicenter Clinical Randomized Controlled Trial Evaluation of an Implant System Designed for Enhanced Primary Stability. Int. J. Oral Maxillofac. Implant..

[19] Lakha T., Kheur M., Hammerle C., Kheur S., Qamri B. (2023). Comparative Evaluation of Marginal Bone Levels, ISQ Trends, and Implant Survival Rates Between Conventional Drilling and Osteotome Technique Using Implants of Varied Lengths: A Split-Mouth Randomized Controlled Clinical Trial. Int. J. Prosthodont..

[20] Patel A., Gil L.F., Castellano A., Freitas G., Navarro D., Peredo A.P., Tovar N., Coelho P. (2016). Effect of Simplified One-Step Drilling Protocol on Osseointegration. Int. J. Periodontics Restor. Dent..

[21] Ercoli C., Funkenbusch P.D., Lee H.J., Moss M.E., Graser G.N. (2004). The influence of drill wear on cutting efficiency and heat production during osteotomy preparation for dental implants: A study of drill durability. Int. J. Oral Maxillofac. Implant..

[22] Yacker M.J., Klein M. (1996). The effect of irrigation on osteotomy depth and bur diameter. Int. J. Oral Maxillofac. Implant..

[23] Benington I.C., Biagioni P.A., Briggs J., Sheridan S., Lamey P.J. (2002). Thermal changes observed at implant sites during internal and external irrigation. Clin. Oral Implant. Res..

[24] Musskopf M.L., Finger Stadler A., Fiorini T., Ramos U.D., de Sousa Rabelo M., de Castro Pinto R.N., Susin C. (2024). Performance of a new implant system and drilling protocol-A minipig intraoral dental implant model study. Clin. Oral Implant. Res..

