# Supplementary material for: Bone Remodeling and Marginal Bone Loss of Simplified Versus Conventional Drilling: A Randomized Clinical Trial
Source: Bioengineering (Basel). 2025 Feb 13;12(2):178. doi: 10.3390/bioengineering12020178 (PMC11852080; doi:10.3390/bioengineering12020178)
Supplement: Supplementary file 1 [file bioengineering-12-00178-s001.zip › bioengineering-3396695-supplementary.pdf]

Table S1. Multiple lineal regression models using MBL (Baseline-24 month) as primary outcome.

Model	Parameter	Estimate	Std.Error	Beta	t	p-value
Avarage_MBL p-value=.130 Adjusted R^2 =.0509	Constant	-2.181	1.086		-2.008	0.052
	Group	0.095	0.174	0.083	0.545	0.589
	Implant position	0.010	0.18	0.209	0.580	0.566
	Sex	-0.407	0.183	-0.352	-2.231	0.032
	Implant diameter	0.442	0.223	0.328	1.986	0.055
	Implant length	0.067	0.073	0.136	0.920	0.364
	Arch	-0.205	0.405	-0.176	-0.506	0.616
	Tooth	0.053	0.234	0.043	0.225	0.823
